# A comparison of two studies and the prevalence and sex ratio of Neurodevelopmental conditions in Tuberous Sclerosis Complex

**DOI:** 10.1186/s13023-021-01984-1

**Published:** 2021-08-18

**Authors:** Abigail K. Runicles, Charlotte Tye, Patrick F. Bolton

**Affiliations:** 1 Department of Child and Adolescent Psychiatry, Institute of Psychiatry Psychology and Neuroscience, King’s College London, London, UK; 2 St Georges University of London, Cranmer Terrace, Tooting, London, UK; 3 Department of Psychology, Institute of Psychiatry Psychology and Neuroscience, King’s College London, London, UK; 4 Social, Genetic and Developmental Psychiatry Centre, Institute of Psychiatry, Psychology and Neuroscience, King’s College London, London, UK

Various forms of psychopathology have been associated with Tuberous sclerosis complex (TSC) including autism spectrum disorder (ASD), attention deficit hyperactivity disorder (ADHD) and intellectual disability (ID) [1, 2], but prevalence estimates have varied widely in studies. The TOSCA (TuberOus SClerosis registry to increase disease Awareness) international disease registry study first described rates of TSC Associated Neuropsychiatric Disorders (TAND) in a large sample size of 2216 participants in 2018 [3]. More recently the TOSCA team [3] have reported further results on TAND data from the TOSCA study [4]. They report prevalence rates, as well as sex (male preponderance of ASD and ADHD) and age differences (higher rates of ADHD in children) in the prevalence of ASD and ADHD. The authors acknowledge various methodological limitations (ascertainment bias, reliance on clinical diagnosis rather than systematic standardised evaluation and very high (60%) rates of missing data), but they suggest that these methodological limitations are partly offset by the large-scale “real world” nature of their study. In our view, the nature and extent of the methodological limitations in the study may not be fully appreciated by readers. We highlight the potential impact of these methodological shortcomings by contrasting the TOSCA TAND results with findings from a high quality, methodologically rigorous prospective longitudinal study of a general population representative sample of individuals with TSC—the Tuberous Sclerosis 2000 (TS 2000) study (1,3,4,6,8, 12–14). In the first phase of the TS 2000 study, 125 children with TSC (63 females, 62 males; median age = 39mo) were recruited. In the second phase of the study, at an average of 8 years later, neurodevelopmental outcomes were assessed with 88 participants (median age = 148 mo) (1,3,4,6,8, 12–14).

First, we discuss significant methodological considerations for studies determining prevalence of psychopathology in TSC:

1. TOSCA study participants were recruited through attendance at a variety of participating specialist TSC clinics either at the time of diagnosis of TSC or if they had an established diagnosis of TSC and attended a clinic for this in the previous 12 months. This means that ascertainment will be biased in favour of cases of TSC with clinical (including behavioural and psychiatric) complications and the reason for clinic attendance is likely to have differed in adults and children;
2. Cases were recruited from 170 sites, across 31 countries [3] and the presence and type of TAND was based on retrospective review of clinical records using the TAND checklist, but no formal evaluations of TAND were mandated in the study [3]. While it is important to determine global prevalence rates to understand the impact of geographic, cultural/ethnic and socioeconomic factors, inequalities in the clinical identification and diagnosis of TAND is well recognised because of marked differences in the nature of health care systems and diagnostic practices between countries (including utilisation of different diagnostic nosological systems which have evolved over time).'
3. Operational criteria for assigning a diagnosis of ASD and ADHD are not provided in the TAND Checklist, and inter-rater reliability for assigning diagnoses has not been reported in the TOSCA study. Moreover, the patients attended a wide range of different types of clinics (genetic, paediatric, epilepsy, etc.,) that were led by specialists from many different disciplines with variable levels of expertise in clinically recognising and diagnosing TAND. In the TS 2000 Study, gold standard diagnostic tools were combined with other measures (e.g. the development and well-being assessment), and a best estimate clinical diagnosis of either definite or probable ASD and ADHD was assigned by a psychiatrist. Furthermore, the diagnostic criteria for ADHD require evidence for pervasiveness across situations (e.g. present in home and school) as well as symptoms to have commenced in early childhood, so good quality developmental information measuring behaviour at home and school is important. Lastly, different thresholds for diagnosis of ASD and ADHD traits make it challenging to determine true prevalence;
4. These issues may have been further compounded by the language differences between centres and potential for diagnostic ‘over shadowing’ (i.e. TAND being overlooked because of clinical focus on physical complications);
5. The TOSCA study ascertained participants with a wide age range (< 1–71 years of age) and report diagnostic and behavioural data in very young children as well as older adults, in whom the clinical identification and diagnosis of TAND is recognised to be very challenging and diagnostic reliability and stability is known to be low. Similarly, misdiagnosis in females is another potential bias in the TOSCA study, as studies have consistently shown that there is a diagnostic gender bias in neuro-developmental disorders and females are at a disproportionate risk of not receiving a clinical diagnosis [5].

These methodological issues may have led to significant bias and misleading findings and therefore the results should be interpreted with caution. To illustrate the impact of these limitations, we contrast the TOSCA findings with findings from the TS 2000 cohort study. The TS 2000 study is a UK population-based, prospective longitudinal study of the natural history of TSC. It was designed to prospectively track the natural history of TSC following initial diagnosis and chart the emergence of complications, including TAND. Cases in the TS 2000 study were typically diagnosed in infancy due to the physical or neurological manifestations such as epilepsy. Psychopathology was comprehensively and systematically assessed using well-established and validated diagnostic tools once participants had reached 6 + years of age, by which time the assessment of TAND is valid [1]. While having a smaller sample size to TOSCA, identifying cases at first diagnosis before they develop neuro-psychiatric problems and then following up over time gives a more accurate estimate of the prevalence, correlates and of co-morbidities in psychopathology in the TSC population [6].

The prevalence estimates of ASD, ADHD and ID in the TS 2000 and TOSCA studies are shown in Table 1 [1, 3, 7–9]. There are significant and substantial differences in estimates of ASD and ADHD with rates being significantly higher in the TS 2000 study compared to the TOSCA study, but no differences in ID between the studies. Arguably, the prevalence of ID did not differ because the identification and diagnostic tools and criteria for ID are fairly uniform and therefore more straightforward to assess across countries. By contrast, complex psychopathologies such as ASD and ADHD where diagnostic tools and operational criteria are increasingly recognised as necessary aids to clinical diagnosis, show quite significant differences in prevalence estimates between studies. Moreover, the findings from the TS 2000 study regarding the prevalence of definite and probable ASD and ADHD demonstrate how sensitive estimates are to variations in where the threshold for diagnosis is placed, when dealing with dimensionally distributed traits.

**Table 1:** Prevalence of ASD, ADHD and ID in the TS 2000 and TOSCA studies

	TS 2000—prevalence	TOSCA—prevalence	Chi-squared analysis
Definite ASD^a^	43% (n = 80)	21% (n = 1486)	${ X}^{2}$(1) = 18.84***
Probable ASD^b^	68% (n = 80)	–	${X}^{2}$(1) = 88.23***
Definite ADHD^c^	23% (n = 78)	19% (n = 1404)	${ X}^{2}$(1) = 0.52
Probable ADHD^d^	46% (n = 78)	–	${ X}^{2}$(1) = 31.56***
Normal IQ (70+)	43% (n = 88)	44% (n = 885)	${ X}^{2}$(1) = 0.01
Mild-to-moderate intellectual disability (35–70)	45% (n = 88)	43% (n = 885)	${ X}^{2}$(1) = 0.09
Severe-to-profound intellectual disability (0–34)	11% (n = 88)	12% (n = 885)	${X }^{2}$(1) = 0.01

*P ≤ 0.05, **P ≤ 0.01, ***P ≤ 0.001

^a^ADI-R and ADOS-2 positive

^b^ADI-R or ADOS-2 positive

^c^Pervasive and persistent ADHD

^d^Pervasive or persistent ADHD

Table 2 shows the prevalence of ASD and ADHD in males and females across the TS 2000 and TOSCA studies. In the general population, a large scale general population sample has shown an estimated ratio of males to females of 4.32:1 [5]. Table 2 shows differences between the TOSCA and TS 2000 studies in sex ratio, with no evidence for a sex difference in prevalence of ASD in the TS 2000 study. This raises the possibility that, females were ‘flying below the clinical diagnostic radar’ for a diagnosis of ASD in TOSCA. Interestingly, there was evidence for sex differences in prevalence of ADHD within both the TS 2000 and TOSCA studies [4, 7–9]. However, it should be noted that the sex difference appears to be reduced compared to that observed in general population samples of ADHD; in a community based population, samples have an estimated ratio of males to females close to 2.38:1 [10].

**Table 2:** Male and female prevalence of ASD and ADHD in the TS 2000 and TOSCA studies

	Female prevalence	Male prevalence	Chi-squared analysis
TOSCA (n = 786)			
ASD	13.5%	28.9%	${ X}^{2}$(1) = 27.15***
TS 2000 (n = 80)			
Definite ASD^a^	40.5%	45.9%	${X}^{2}$(1) = 0.07
Probable ASD^b^	75.7%	61.9%	${X}^{2}$(1) = 1.15
TOSCA (n = 752)			
ADHD	16.7%	28.1%	${ X}^{2}$(1) = 13.45***
TS 2000 (n = 78)			
Definite ADHD^c^	15.5%	33.3%	${ X}^{2}$(1) = 2.46
Probable ADHD^d^	35.6%	60.6%	${ X}^{2}$(1) = 3.85*

*P ≤ 0.05, **P ≤ 0.01, ***P ≤ 0.001

^a^ADI-R and ADOS-2 positive

^b^ADI-R or ADOS-2 positive

^c^Pervasive and persistent ADHD

^d^Pervasive or persistent ADHD

The TOSCA study included a wide age range of participants and difference in prevalence estimates in children and adults have been noted by the authors [4]. Table 3 gives prevalence estimates of ASD and ADHD in the TOSCA children compared with the TS 2000 study who were almost all children [4, 7–9]. The findings indicate that the age differences between the cohorts had little effect and this cannot account for the different prevalence estimates in the studies.

**Table 3:** Characteristics of ASD and ADHD in the child and adolescent population in the TS 2000 and TOSCA studies

	TS 2000—prevalence	TOSCA—prevalence	Chi squared analysis
Child and adolescent			
Definite ASD^a^	43% (n = 80)	22% (n = 586)	${X}^{2}$(1) = 15.54***
Probable ASD^b^	68% (n = 80)	–	${X}^{2}$(1) = 72.40***
Child and adolescent			
Definite ADHD^c^	23% (n = 78)	25% (n = 571)	${X}^{2}$(1) = 0.07
Probable ADHD^d^	46% (n = 78)	–	${X}^{2}$(1) = 13.98***

*P ≤ 0.05, **P ≤ 0.01, ***P ≤ 0.001

^a^ADI-R and ADOS-2 positive

^b^ADI-R or ADOS-2 positive

^c^Pervasive and persistent ADHD

^d^Pervasive or persistent ADHD

In summary, while the TOSCA study has a uniquely large sample size, it is subject to methodological limitations that have likely significantly influenced the study findings. We therefore emphasise the critical need for future research to use the TAND checklist in combination with gold-standard diagnostic tools and rigorous methodological study design.

## Data Availability

The datasets used and/or analysed during the current study are available from the corresponding author on reasonable request.

## Abbreviations

ADHD: Attention Deficit Hyperactivity Disorder
ASD: Autism Spectrum Disorder
ID: Intellectual Disability
IQ: Intelligence Quotient
TAND: TSC-Associated Neuropsychiatric Disorders
TOSCA: TuberOus SClerosis registry to increase disease Awareness
TSC: Tuberous Sclerosis Complex
TS 2000: Tuberous Sclerosis 2000 Study
